# Polyethersulfone mixed matrix membranes modified with pore formers and Ag-titanate nanotubes: physicochemical characteristics and (bio)fouling study

**DOI:** 10.1007/s11356-024-35461-6

**Published:** 2024-11-08

**Authors:** Amanda Sałacińska, Paulina Sienkiewicz, Kacper Szymański, Sylwia Mozia

**Affiliations:** Department of Inorganic Chemical Technology and Environment Engineering, Faculty of Chemical Technology and Engineering, West Pomeranian University of Technology in Szczecin, Ul. Pułaskiego 10, 70 – 322 Szczecin, Poland

**Keywords:** Polyvinylpyrrolidone, Poly(ethylene glycol), Titanate nanotube, Silver, *Escherichia coli*

## Abstract

In the presented studies it was hypothesized that the modification of a polymeric membrane with a pore former and a hybrid nanomaterial composed of titanate nanotubes with deposited Ag nanoparticles (Ag-TNTs NPs) can protect the membrane from the microbial growth, and thus enhance its resistance to biofouling. Polyethersulfone (PES) membranes were prepared by the wet phase inversion, and polyvinylpyrrolidone (PVP) and poly(ethylene glycol) (PEG) were used as pore formers. The membranes were characterized in terms of morphology, topography, permeability, separation characteristics, and anti-(bio)fouling properties as well as antibacterial activity. The membranes modified with porogens and Ag-TNTs revealed improved hydrophilicity and water permeability compared to the unmodified membrane, from 58 to 66%. Moreover, the improvement in rejection of model dextrans and PEG upon application of the NPs was found. However, the use of PVP or PEG had a negative influence on the resistance to fouling by bovine serum albumin, i.e., ca. 35% of decline of permeate flux was noticed after 2 h of ultrafiltration of BSA. On the contrary, both porogens and NPs contributed to biofouling mitigation. The introduction of pore formers had a positive effect on the inhibition of *Escherichia coli* growth by the membrane containing Ag-TNTs. The log reduction of bacteria varied from 3.17 to 3.3 in case of stirred and filtration system.

## Introduction

Mixed matrix polyethersulfone (PES) membranes are widely used in ultra- and microfiltration processes, however, the main drawback of their usefulness is their susceptibility to (bio)fouling due to relatively low hydrophilicity. Therefore, many various modifications of such membranes were proposed (Otitoju et al. 2018; Kahrs and Schwellenbach 2020). The simplest approach is to add some modifying agent into dope solution and mixing them together. As a result, the obtained membranes have different properties. The pore size distribution, the thickness and topography of top layer of membrane, hydrophilicity and surface charge are the most important factors affecting the performance and resistance to (bio)fouling of modified membrane. These parameters can be controlled at the stage of preparation of casting dope, especially by selecting polymer and presence of additives (Marchese et al. 2003; Otitoju et al. 2018; Kahrs and Schwellenbach 2020). Hydrophilic polymers are used to fabricate the membranes with higher porosity, permeability and hydrophilicity. Very often as a pore-forming agents in the preparation of mixed matrix membranes (MMMs), polyvinylpyrrolidone (PVP) and poly(ethylene glycol) (PEG) are used (Abdel-Karim et al. 2017; Khalaf et al. 2017; Kahrs and Schwellenbach 2020; Mokarizadeh and Raisi 2021). The influence of these additives was widely studied in terms of morphology and hydrophilicity (Aminudin et al. 2017; Malik et al. 2019; Rajabi et al. 2020). The addition of PVP and PEG to casting dope resulted in increasing its viscosity and decreasing of miscibility with the non-solvent (Han and Nam 2002), what affected the final structure and performance of the fabricated membrane (Sadrzadeh and Bhattacharjee 2013). According to Sadrzadeh et al. (Sadrzadeh and Bhattacharjee 2013) the addition of PVP and PEG in various concentrations and molecular masses caused formation of the membranes with sponge-like structure due to reducing the demixing rate. Vatsha et al. (Vatsha et al. 2014) during investigations on PES membranes modified with PVP (40 kDa) 10 wt.% revealed that the dope solution containing 16 wt.% PES led to the creation of membranes with higher pure water flux. Several researchers reported a positive effect of the addition of both porogenes on the membrane fouling alleviate (Rabiller-Baudry et al. 2015; Mokarizadeh and Raisi 2021; Vaysizadeh et al. 2021; Li et al. 2021; Febriasari et al. 2021). This was caused by improvement of hydrophilicity of obtained membrane, thereby reducing the adsorption of macromolecules onto membrane surface. Additionally, PEG could form a thin hydration layer, which prevented the deposition of pollutants, e.g., proteins (Lowe et al. 2015). In turn, the antifouling properties of PVP result from the formation of water barrier by polar pyrrolidone units (Zhang et al. 2015).

In case of mixed matrix membranes modified with nanoparticles, the PVP and PEG act as dispersant, stabilizers or/and binders (Zhang et al. 2015; Warsinger et al. 2018; Hashim et al. 2018; Ursino et al. 2018; Jaleh et al. 2020). Additive of NPs causes improvement of membrane hydrophilicity, resulting in better water permeance and antifouling properties (Padaki et al. 2015). TiO_2_, SiO_2_, Al_2_O_3_, ZrO_2_, ZnO, carbon nanotubes (CNTs), titanate nanotubes (TNTs), and halloysite nanotubes (HNTs) have been mostly used as nanofillers (Otitoju et al. 2018; Szymański et al. 2021; Calabrese et al. 2022). However, the main drawback of such inorganic nanomaterials is low affinity to the polymer matrix, therefore their distribution in the membrane matrix is usually uneven. To address this issue, some researchers have studied the relationship between pore-forming agents, NPs and membrane properties. For instance, Zhang et al. (2015) observed better dispersion of TiO_2_ particles in the polyvinylidene difluoride (PVDF) membrane structure resulted from the steric hindrance effect of PEG chains adsorbed on the TiO_2_. The hydrophilic properties of PEG can also improve the dispersion of TiO_2_ in the membrane structure due occurrence of the coordination sites for NPs through hydrogen bonds (Zhang et al. 2022). Nevertheless, it was found, that the PEG hindered the agglomeration of TiO_2_ particles solely at low concentration (< 0.15 wt.%) (Zhang et al. 2022). Generally, the increasing of nanomaterials amount in the casting dope affects the presence of aggregated NPs in membrane matrix (Zhang et al. 2015). Garcia-Ivars et al. (2014) noted that addition of two hydrophilic modifiers, i.e., PEG (0.4 kDa) and Al_2_O_3_, to the PSU, PES, and polyetherimide (PEI) membrane improved their hydrophilicity; however, for PEG content above 2 wt.% in the membrane structure, macrovoids were formed, negatively affecting the antifouling properties of obtained membranes.

Despite the fact that the additive of both polymers, PVP and PEG, together with inorganic nanofillers improves the hydrophilicity, permeability as well as resistance to fouling of MMMs, the reports on the impact of such modifications on the antimicrobial properties and the resistance to fouling of prepared membranes are very limited. Our research group proposed the application of titanate nanotubes as the promising nanofillers that improved the antibacterial properties of PES mixed matrix membranes (Mozia et al. 2021). In order to further improve the antimicrobial properties of TNTs and reduce the formation of biofilm onto membrane surface, the modifying of the nanoparticles with well-established antimicrobial agents, silver or copper, were studied (Szymański et al. 2020, 2021). It was found that the presence of Ag-TNTs or Cu-TNTs in the matrix of PES membranes improved their desired properties such as permeability, resistance to contamination and antimicrobial performance.

In the present research, we focused on the effects of PEG and PVP as hydrophilic organic additives and Ag-TNTs as an inorganic nanofiller characterized by hydrophilic and antibacterial properties, on the performance of ultrafiltration mixed matrix PES membranes. The reason to perform these studies was the possibility of mitigating membrane (bio)fouling by proposed modification of MMMs. The physicochemical, separation, anti-(bio)fouling and antibacterial properties of the modified membranes are particularly described and discussed. Two experimental systems, i.e., a mixing system and a filtration system, were applied to evaluate the antimicrobial activity of prepared membranes, their resistance to biofouling, as well as the quality of the permeate.

The literature reports cited in Table 1 revealed that researchers added various types of silver nanoparticles and assessed most of all the antibiofouling properties (caused by bacteria) of the modified membranes or added PVP or PEG or another hydrophilic additive (e.g., PVA) and determined the antifouling properties (caused by organic substances) of the membranes. In the presented work, a synergistic approach was proposed, i.e., the effect of PVP, PEG, and Ag nanoparticles on both antibacterial and antibiofouling properties was investigated. This issue distinguishes the presented studies from the previously published reports (Table 1) and emphasizes the novelty of the proposed solution.

**Table 1:** Summary of the polyethersulfone mixed matrix membranes of various modifications

No	Modification technique	Nanofiller	Antibacterial properties	Fouling	Ref
1	Blending nanoparticles with polymer	Silver nanoparticles (AgNPs)–halloysite nanotubes (HNTs)–reduced graphene oxide (rGO) nanocomposite (AgNPs–HNTs–rGO)	Ideal bacteriostasis rate against *E. coli* even after six months of storage	Flux decline caused by BSA less significant, chemical cleaning effectively recovers the membrane	Zhao et al. (2015)
2	Incorporation via phase inversion technique	Multi-walled carbon nanotubes (MWCNTs) coated with silver nanoparticles (AgNPs)	Long term antibacterial activity nanocomposite membranes against *E. coli* and *S. aureus*, higher inhibition towards* E.coli*	Low fouling self-cleaning properties	Al Aani et al. (2017)
3	Incorporation via phase inversion technique	Silver nanoparticles (AgNPs)	Maximum reduction of *E.coli* (66%) for membrane containing 1.5% of AgNPs	Self-cleaning properties	Rana et al. (2017)
4	Blending of functional nanocomposites with polymer	Ag-polydopamine (Ag-PDA) particles	Less *E. coli* attachment into membrane surface	No fouling, > 99% dye rejection	Maganto et al. (2021)
5	Blending with polymer, polyvinylpyrrolidone	Zwitterionic graphene oxide (GO-Arg) nanostructures	Almost complete *E. coli* inhibition after 24 h	Reversible fouling higher for modified GO-embedded membranes, irreversible fouling higher for PES and PES/PVP membranes	Espinoza Castellanos et al. (2024)
6	Non-solvent induced phase separation method	ZIF-8 (metal–organic framework obtained by mixing Zn(NO_3_)_2_·6H_2_O with C_4_H_6_N_2_ and TiO_2_)	-	27.8% of total BSA fouling rate due to higher hydrophilicity of membrane	Zhang et al. (2024)
7	Embedded into membrane via phase inversion process	Ti_3_SiC_2_ and Ag-modified Ti_3_SiC_2_, polyvinyl alcohol	-	The highest antifouling performance (BSA) for 0.75 wt% Ag-Ti_3_SiC_2_	Tizhoush et al. (2024)
8	Non-solvent induced phase separation technique	Triethylene glycol (TEG), polyethylene glycol (PEG), polyvinylpyrrolidone (PVP)	-	Improved antifouling performance of the membrane towards oil droplets from refinery and textile industrial wastewaters	Mokarizadeh and Raisi (2021)
9	Phase inversion method	Al_2_O_3_ and PEG	-	Superior antifouling properties for PEG400 additive less than 2 wt% due to higher hydrophilicity of membrane	Garcia-Ivars et al. (2014)

## Experimental

### Materials

Polyethersulfone (Ultrason E6020P) was obtained from BASF SE (Germany). *N,N –* dimethylformamide (DMF) (puriss p.a.) and AgNO_3_ (puriss p.a.) were purchased from Avantor Performance Materials Poland S.A. (Poland). Bovine serum albumin (BSA, Probumin) was purchased from Merck (Germany). PEG with molecular weight of 4, 10, 20, and 35 kDa, and PVP with molecular weight of 10 kDa were obtained from Sigma-Aldrich (USA). Dextrans with molecular weight of 70, 110, 200, and 500 kDa were provided by Polfa Kutno (Poland). AEROXIDE® TiO_2_ P25 was obtained from Evonik Industries (Germany). The 10 kDa PEG and 10 kDa PVP were applied as pore-forming agents during membranes fabrication. The 4, 20, and 35 kDa PEG as well as dextrans were used for the determination of separation properties of the membranes.

In the microbiological tests the plate count agar (PCA, BIOCORP, Poland) and NaCl (puriss p.a., Merck, Germany) were applied. The NaCl solution was prepared by dissolution of 8.5 g of the chemical in deionized water followed by sterilization. The PCA solution was prepared according to the instruction given by the manufacturer and then poured into Petri dishes. The dishes were subsequently sterilized under UVC irradiation for 20 min and dried for 3 days at 37 °C.

The Gram-negative *Escherichia coli* strain (ATCC 29425) was used as a model microorganism. The concentration of bacteria suspension in NaCl solution was set at 0.5 or 0.1 according to the McFarland scale (McFarland standards, bioMérieux, France).

In all experiments deionized water from Elix 3 (Millipore, USA) was applied.

### Preparation of nanomaterials

#### Preparation of titanate nanotubes

TNTs were prepared using hydrothermal approach (Mozia et al. 2021). In brief, TiO_2_ (2 g) and 10 mol/L NaOH solution (60 mL) were introduced into a Teflon vessel and then dispersed for 1 h at room temperature in an ultrasonic bath. Subsequently, the vessel was placed in an autoclave and heated at 140 °C for 24 h. Then the mixture was washed with 0.1 mol/L HCl and deionized water. The obtained TNTs were dried at 80 °C for 12 h.

#### Preparation of TNTs modified with silver

Silver nanoparticles were deposited on TNTs by photoreduction. The TNTs (1 g) were introduced into a glass reactor filled with 0.1 mol/L AgNO_3_ solution (100 mL). The mixture was dispersed using a magnetic stirrer (250 rpm) for 2 h. While stirring continuously, the suspension was irradiated with a low-pressure mercury lamp (TNN 15/32, Heraeus Noblelight GmbH, Germany, 15 W, λ_max_ = 254 nm) for 2 h. Subsequently the suspension was centrifuged and washed several times with deionized water to completely remove the excess Ag^+^ ions. The obtained Ag-TNTs nanoparticles were dried at 80 °C for 12 h. Detailed characteristics of Ag-TNTs can be found elsewhere (Jose et al. 2019).

### Preparation of membranes

The PES UF membranes were prepared by the wet phase inversion method. In the case of the unmodified membrane (UM) the polymer (15 wt.%) was dissolved in DMF (85 wt.%). The homogeneous dope solution was casted onto a glass plate using an automatic film applicator (Elcometer 4340, Elcometer Ltd., UK) with the knife gap of 0.1 mm, and subsequently immersed in a pure water bath (type 2, 0.066 µS/cm, Millipore, 20 ± 1 °C) to complete the phase inversion process.

The casting dope for the fabrication of the mixed-matrix membranes was obtained by mixing a dispersion of NPs in DMF (10 mL) with the solution of PES in DMF (40 mL). The dispersion of NPs was prepared using ultrasonic probe (Vibra-cell VCX-130, Sonics, USA). After the addition of the Ag-TNTs dispersion to the PES solution, the casting dope was mixed alternately using: (i) a magnetic stirrer at the temperature of 55–60 °C and (ii) sonication in an ultrasonic bath (Sonic-6D, Polsonic, Poland) for 2 h, 15 min by turns. Subsequently, the membranes were casted as described above. In the case of the membranes modified with the pore-forming agents, the PEG (PESG and PESG/Ag-TNT membranes) or PVP (PESP and PESP/Ag-TNT membranes) were introduced into the PES solution. The molecular weight of PEG and PVP was 10 kDa. The composition of the casting dope is presented in Table 2.

**Table 2:** Composition of casting dope applied for membrane preparation

Membrane	PES [wt.%]	DMF [wt.%]	PEG 10 kDa [wt.%]*	PVP 10 kDa [wt.%]*	Ag-TNTs [wt.%]**
UM	15	85	0	0	0
PES/Ag-TNT	15	85	0	0	1
PESP	15	84	0	1	0
PESP/Ag-TNT	15	84	0	1	1
PESG	15	84	1	0	0
PESG/Ag-TNT	15	84	1	0	1

^*^By mass of the solution

^**^By mass of the polymer

### Characterization of membranes

#### Physicochemical properties, topography and morphology

Surface topography of the membranes was analyzed using atomic force microscope (NanoScope V Multimode 8, Bruker Corp., USA) in the ScanAsyst mode with the application of a silicon nitride probe. The average roughness value (R_a_) was determined in the NanoScope Analysis software based on 10 µm × 10 µm AFM images. The morphology of the membranes cross section was studied using the ultra-high-resolution field-emission scanning electron microscope (UHR FE—SEM) Hitachi SU8020 (Germany). The samples of membranes were dehydrated in ethanol, broken in liquid nitrogen, and sputtered with chromium (Q150T ES Quorum Technologies Ltd., UK). The analysis was carried out using secondary electrons (SE) mode at the accelerating voltage of 5 kV. Static contact angle (SCA) of the membranes was determined by the sessile drop method using a goniometer (type 260 ramé-hart instruments co., USA).

#### Permeability, fouling resistance and separation properties of membranes

Pure water flux was evaluated by ultrafiltration (UF) of deionized water (20 °C) at a transmembrane pressure TMP = 1, 2, and 3 bar. The membrane fouling was examined with application of BSA solution (1 g/L) during 2 h of UF process (cross flow velocity = 1 m/s and TMP = 2 bar). Separation properties were evaluated by ultrafiltration of model solutions (0.5 g/L, TMP = 1 bar) containing PEGs (4, 20, 35 kDa) and dextrans (70, 110, 200, 500 kDa). Each experiment was repeated at least three times to confirm reproducibility of the results.

The rejection of PEGs and dextrans was calculated based on Eq. (1):

1 $$\text{R}=\frac{{C}_{f}- {C}_{p}}{{C}_{f}}\times 100\%$$

where R is the rejection coefficient, C_f_ is the concentration of model compound in feed, and C_p_ is the concentration of model compound in permeate.

The concentration of PEGs and dextrans was measured using high-performance liquid chromatograph (HPLC) LaChrom Elite (Hitachi, Japan) equipped with the refractive index (RI) detector L-2490 and the PolySep-GFC-P4000 column (Phenomenex, USA). Ultrapure water was used as a mobile phase.

#### Antibacterial properties

Antibacterial properties of the membranes were determined using two approaches. The first method was based on stirring tests (stirred system). The membrane samples (12.5 cm × 4.5 cm) were dehydrated in ethanol, dried in air and immersed in 0.1 L of *E. coli* solution (optical density of 0.5 according to the McFarland scale). The control sample was prepared without the membrane. The samples were incubated at 37 °C for 24 h with a continuous mixing using a magnetic stirrer (250 rpm). Then the bacteria were counted using the serial decimal dilutions method. A total of 0.3 ml of diluted solution was spread on the agar plate, which was subsequently incubated at 37 °C for 24 h. The bacteria colonies were calculated by the counter (LKB 2002, POL-EKO, Poland). The average colony forming unit (CFU) was determined according to Eq. (2):

2 $$\text{CFU}/\text{ml}=\frac{\text{N }\times \text{ Y}}{\text{Z}}$$

where N is the number of bacteria colonies visible on agar plates, Y is the dilution factor, and Z is the volume of bacteria suspension on Petri dish (0.3 mL).

The log reduction of bacterial growth was determined using Eq. (3):

3 $$\log\;reduction=\log\;\left(\frac AB\right)$$

where A is the amount (CFU/mL) of bacteria in control sample, and B is the amount (CFU/mL) of bacteria in the presence of a membrane.

The second method of determination of the antibacterial properties was based on the filtration of bacteria suspension (filtration system). In this case, a cross flow UF installation equipped with two stainless steel membrane modules (with a 1.194-mm feed spacer), a peristaltic pump with two heads, two-needle valves with manometers, and a feed tank, was used. The working area of each membrane was 0.0025 m^2^. The feed constituted a suspension of *E. coli* bacteria in NaCl solution with an optical density of 0.1 (according to the McFarland scale). The feed was incubated at 37 °C. The feed cross flow velocity was 0.25 m/s and the transmembrane pressure was 1 bar. The UF was carried out for 6 h. The samples of the permeate and feed were collected every 2 h. Each experiment was repeated at least three times to confirm reproducibility of the results.

## Results and discussion

### Physicochemical properties of membranes

**Fig. 1:**
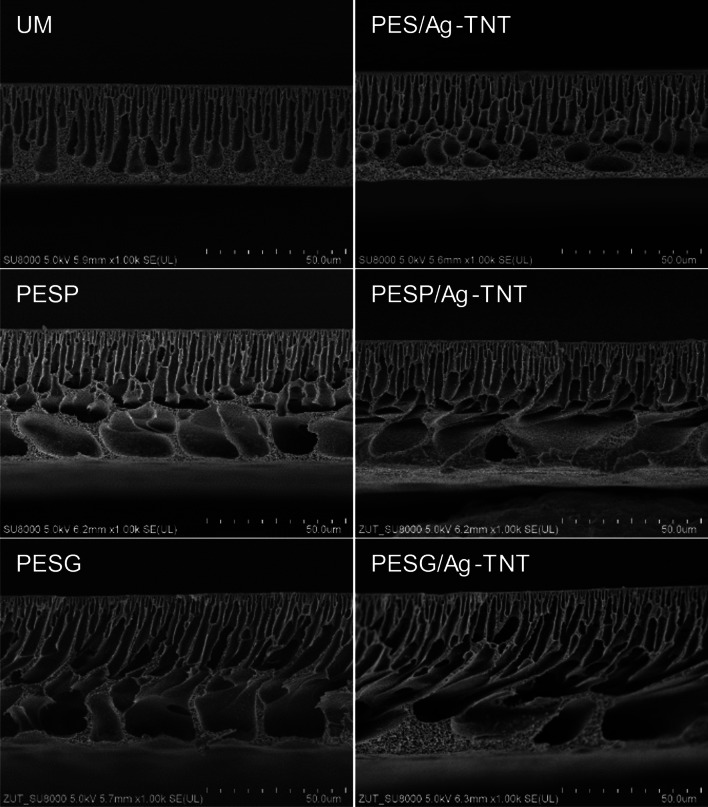
Influence of the pore-forming agents and Ag-TNTs on SEM cross sections of the PES membranes

Morphology of the membranes was examined on a basis of SEM images shown in Fig. 1. The membranes prepared without addition of the pore-forming agents exhibited an asymmetric structure with a thin separation layer in the upper part, finger-like pores in the middle and a spongy structure in the bottom of the cross section. The addition of the pore-forming agents changed the morphology of the membranes in comparison to the unmodified UM. The introduction of both PVP and PEG to the casting dope resulted in a formation of finger-like pores in the upper part of the membrane, and large, irregular macrovoids with a spongy structure between them in the bottom part of the cross section.

PVP at low concentration enhances the rate of solvent-nonsolvent exchange during the phase separation, thus leading to the enlargement of macrovoids and improvement in membrane permeability (Han and Nam 2002; Mosqueda-Jimenez et al. 2004). However, at high PVP content the demixing is delayed and the formation of macrovoids is suppressed. This leads to a lower permeability of membranes. The reason for the different effect of PVP at its various content in the casting dope was explained by the reduced miscibility of the casting dope with a nonsolvent (water) upon addition of the pore former (thermodynamic enhancement for phase separation) and the simultaneous increase in the dope viscosity (kinetic factor based on rheological hindrance in demixing of the solution acting against phase separation). Depending on the PVP content, one of the factors plays a key role leading to the various membrane structure. The morphology of the membranes presented in Fig. 1 confirms the enhanced demixing rate at low PVP and PEG concentration (1 wt.%, Table 1).

From Fig. 1, it can also be found that the application of the pore-forming agents resulted in an increase in membrane thickness. The values determined on a basis of SEM images changed from ~ 40 µm for the membranes without addition of the porogens to ~ 50 µm for the membranes modified with PVP and ~ 55 µm in the case of the membranes modified with PEG. The difference between the thickness of the membranes with and without addition of the porogens can be attributed to the changes in their porosity. Li et al. (2004) during their investigations on the asymmetric membranes prepared from PES and polyimide found that a so-called “critical structure-transition thickness” exists. An increase in membrane thickness above this value results in the transition of the membrane morphology from a sponge-like to a macrovoid-type structure. Thus, a macrovoid-type structure corresponded to a thicker membrane, which is consistent with the morphology observed in Fig. 1. Mokarizadeh and Raisi (2021) observed the dependence between PEG or PVP additives and the morphology and structure of the PES membranes as well. The results revealed that for various types and concentrations of the additives in the casting solution, PES/PVP membranes had a denser structure than the PES/PEG membranes (Mokarizadeh and Raisi 2021). In turn, according to Rajendaren et al. (2024), PEG 200 was more effective in forming a sponge-like structural wall in the PES membrane than PVP 10000 and reached a membrane porosity of 87.1% (Rajendaren et al. 2024).

**Fig. 2:**
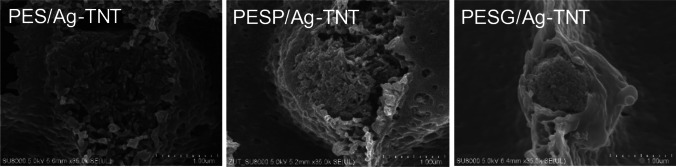
SEM microphotographs of examples of Ag-TNTs agglomerates in the **A** PES/Ag-TNT, **B** PESP/Ag-TNT, and **C** PESG/Ag-TNT membranes

The presence of the NPs did not affect the structure of the membranes, regardless of the synthesis parameters (Fig. 1). Both the cross section morphology and the thickness of the membranes with and without the addition of the nanofiller were similar. Nonetheless, a more detailed analysis (Fig. 2) revealed that the Ag-TNTs form agglomerates with a random size and distribution.

Both small aggregates with diameters below 1 µm, and large agglomerates with diameters reaching even up to 7 µm were found in all the membranes. Nonetheless, most of the diameters ranged from 1 to 5 µm. No clear influence of the PEG or PVP addition on the agglomerate size was found. The agglomeration of Ag-TNTs in the membrane matrix results from the NPs-NPs interactions and the interactions between the NPs and polymer. According to the Derjaguin-Landau-Verwey-Overbeek (DLVO) theory (Derjaguin and Landau 1993; Adamczyk and Weroński 1999), at a sub-nanometer distance between NPs, the contribution of the van der Waals attraction (aggregation factor) is more significant than the electrostatic or static repulsion caused by the overlapping of electrical double layer (dispersion factor). As a result, the formation of clusters is observed, especially at high NPs concentration, due to the reduced interparticle distance. Moreover, rather limited compatibility between most of the polymers applied for membranes fabrication and highly polar inorganic nanoparticles, such as TNTs impedes uniform dispersion of the inorganic additive in a polymer matrix and enhances formation of NPs agglomerates (Yang et al. 2016). The size and dispersion of the NPs agglomerates in the membrane depends also on the viscosity of the dope solution. After introduction of polymers such as PVP or PEG, the pore formers and PES interact and become entangled. As a result, an increase in the solution viscosity is observed (Greenlee and Rentz 2016). During the investigations on the influence of PVP on ZnO NPs dispersion in PVDF membranes it was found (Van den Berg and Ulbricht 2020) that the size of agglomerates was larger in the neat membrane compared to the membrane prepared with the addition of 1 wt.% PVP. That was attributed to a higher viscosity of the dope solution containing the porogen. The authors hypothesized that the agglomeration of the ZnO NPs took place during the phase separation stage. At the increased viscosity of the casting dope the diffusion rate of the NPs was decreased, which limited the size of the formed agglomerates in the membrane prepared with the addition of PVP. However, such a phenomenon was reported for a very high ZnO content (50 wt.%), at which the viscosity of the solution was visibly higher compared to the solution without the nanomaterial or to the casting dope containing 1 wt.% of the NPs (Van den Berg and Ulbricht 2020). In the present work, the content of Ag-TNTs was low and, therefore, did not contribute significantly to the increase in the viscosity of the casting dope. As a result, the sizes of Ag-TNTs agglomerates observed in the various membranes were similar.

**Fig. 3:**
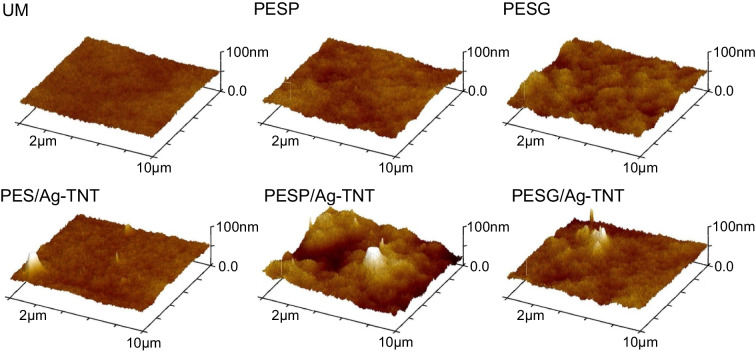
Influence of pore-forming agents and Ag-TNTs on the AFM surface topography of the PES membranes

Membrane topography was investigated based on atomic force microscopy analysis, and the collected images are shown in Fig. 3. In the case of the NPs-modified membranes, the presence of agglomerates and aggregates was observed. On the surface of the PES/Ag-TNT membrane, some small species up to 100 nm were found, but also agglomerates up to 2.5 µm were present. The membranes containing the pore-forming agents were characterized by a more folded structure (Fig. 3).

**Fig. 4:**
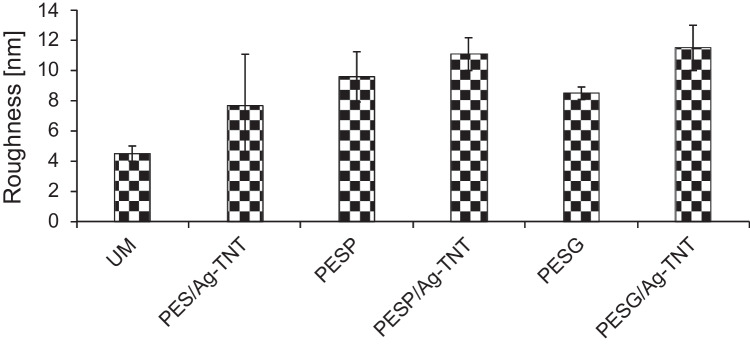
Influence of pore-forming agents and Ag-TNTs on the surface roughness (R_a_) of the PES membranes

Based on the AFM images, the mean surface roughness (R_a_) of the obtained membranes was calculated (Fig. 4). The introduction of PVP and PEG to the PES membrane increased the roughness from 4.5(0.5) nm for UM to 9.6(1.7) nm and 8.5(0.4) nm for PESP/Ag-TNT and PESG/Ag-TNT, respectively. The addition of NPs further increased the roughness to 11.1(1.1) nm for the PESP/Ag-TNT membrane and to 11.5(1.5) nm for PESG/Ag-TNT. After the incorporation of Ag-TNTs in the PES UM membrane, an increase in roughness to 7.7(3.4) nm was observed. The increase in surface roughness upon addition of PVP and PEG is associated with their hydrophilic properties (Vatsha et al. 2014; Hasheminasab et al. 2020). After immersion of the casted polymeric film in the coagulation bath, the porogens leave the membrane matrix by migration from its inside to the outer surface. Since PVP and PEG easily transfer to the surface and separate from it, leaving voids, an increase in roughness is observed compared to the unmodified membrane (Hasheminasab et al. 2020; Lusiana et al. 2020).

**Fig. 5:**
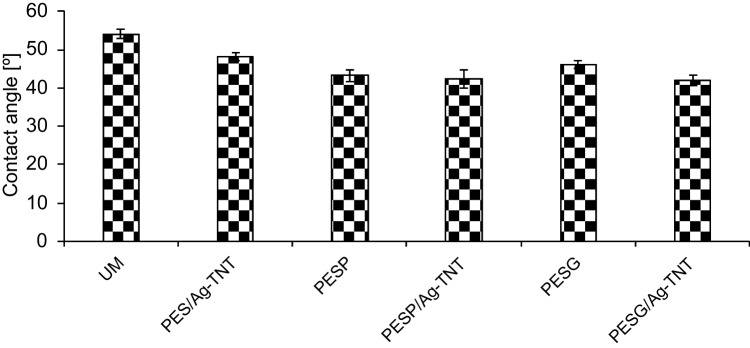
Influence of pore-forming agents and Ag-TNTs on the contact angle of the PES membranes

The introduction of the pore-forming agents into the membrane matrix resulted in a decrease in the contact angle compared to the UM membrane (Fig. 5). However, no significant influence of the type of the pore former on the hydrophilicity was found. The contact angle lowered from 54(1)° for UM to 43(1)° for PESP and 44(4)° for PESG (Fig. 5). Since a complete removal of the porogens during the phase inversion process or even subsequent membrane rinsing is not possible, some residues remain trapped in the membrane matrix (Gebru and Das 2017). Therefore, the observed changes in membrane contact angle resulted from the hydrophilic character of the additives entangled in the membrane.

The application of the Ag-TNTs led to an increase in hydrophilicity of the membranes, regardless of the casting dope composition. The most significant change was observed when no porogens were applied. The contact angle lowered from 54º for UM to 48º for PES/Ag-TNT. In the case of the membranes modified with PVP or PEG the contact angle decreased to 42°, regardless of the pore-forming agent applied (Fig. 5). The changes in the contact angle resulted from the hydrophilic properties of the NPs (Shaban et al. 2015; Padaki et al. 2015; Subramaniam et al. 2018). The hydroxyl groups on the TNTs surface can affect the water attraction to the membrane and, therefore, its improved hydrophilicity is observed (Subramaniam et al. 2018). Moreover, the presence of Ag in the Ag-TNTs can further contribute to the decrease in the contact angle of the membranes. The positive effect of the addition of Ag to the PES membrane on its hydrophilicity was previously reported by other authors (Basri et al. 2011; Zhang et al. 2014). Basri et al. (2011) found that contact angle was reduced from 60° for an unmodified membrane to 51° for a membrane modified with 2 wt.% of silver nanoparticles.

**Fig. 6:**
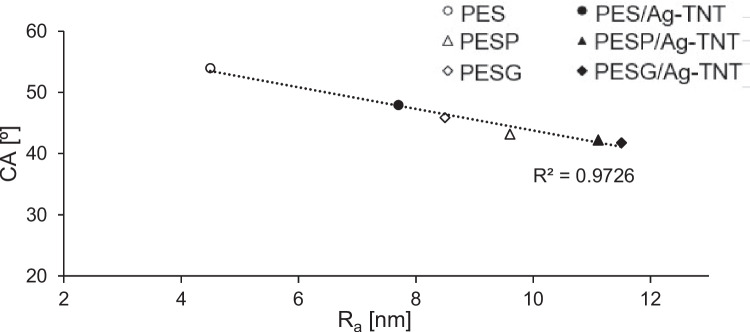
Relationship between water contact angle and surface roughness of the membranes

The lowering of the contact angle of the membranes can be related also to their surface roughness, as can be found from Fig. 6.

In general, when a surface roughness of a hydrophilic material is increasing, a decrease in the contact angle is observed, according to the Wenzel equation (Sotto et al. 2011). Therefore, the observed changes in the membranes hydrophilicity should be treated as a resultant of the hydrophilic properties of the nanomaterial and pore formers, as well as changes in membrane surface roughness being a consequence of the application of the modifiers.

### Permeability of membranes

**Fig. 7:**
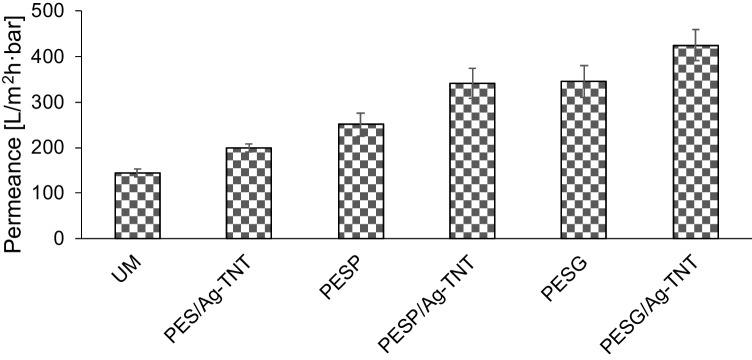
Influence of the Ag-TNTs and pore-forming agents on the permeance of the PES membranes

One of the aims of the modification of the PES membranes with the hydrophilic additives such as PVP/PEG and Ag-TNTs was the improvement in their permeability. The influence of the applied approach on the permeance of the neat and modified membranes is summarized in Fig. 7. It is well established that the transport of water through the membrane is mostly affected by hydrophilicity and porosity of the membrane. In general, hydrophilic and porosity membranes are characterized by higher permeate flux (Ihsanullah et al. 2015).

As was expected, the incorporation of the pore formers resulted in a significant increase in the pure water flux. The most notable effect of the modification was found in the case of PEG. The permeance of the PESG membrane was 2.4 times higher than that of UM membrane and reached 345 L/m^2^h bar. When PVP was applied as a porogen, the permeance increased by 1.7 times and amounted to 252 L/m^2^h bar. The enhancement of membrane permeability after the incorporation of PEG and PVP can be related to the increase in porosity and hydrophilicity. It was previously reported (Wang et al. 1999) that low molecular weight PVP (10–40 kDa) tends to form small pores and is easily washed out from the membrane after the phase inversion, which results in a noticeable flux improvement. Further increase in permeability was observed after the introduction of Ag-TNTs into the membranes (Fig. 7). The permeance of the PES/Ag-TNT, PESP/Ag-TNT and PESG/Ag-TNT was 1.2–1.4 times higher compared to the respective membranes without NPs, and what was 66% and 58% increase, for PESG/Ag-TNT and PESP/Ag-TNT, respectively, in comparison with unmodified membrane (UM). The observed improvement can be attributed to the hydrophilic properties of the nanomaterial, as well as its tubular structure. An increase in membrane permeability was also observed in the case of HNT-chitosan-Ag hybrid nanoparticles (Chen et al. 2013). The abovementioned results proved the synergistic effect of hydrophilic additives – PEG, PVP and Ag-TNTs (Ihsanullah et al. 2015).

### Separation properties

**Fig. 8:**
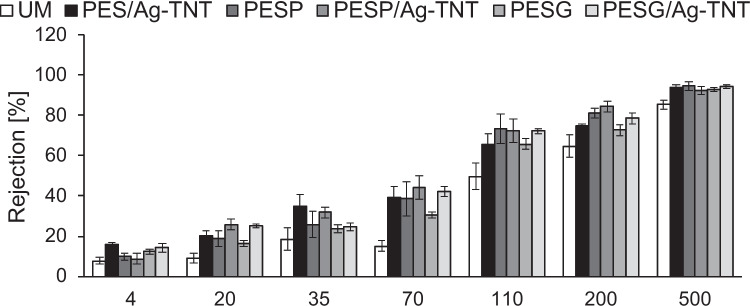
Influence of Ag-TNTs and pore-forming agents on the separation properties of the PES membranes. The molecular weight of 4, 20, and 35 kDa refers to PEG, while 70, 110, 200, and 500 kDa refer to dextrans

Separation properties were determined on a basis of ultrafiltration of model solutions of PEG with molecular weight of 4, 20, and 35 kDa and dextrans with molecular weight of 70, 110, 200, and 500 kDa. The concentration of a model substance was 0.5 g/L. The results are presented in Fig. 8.

The application of the pore-forming agents improved the separation properties of the PES membrane. In the case of the 20 kDa PEG, the rejection coefficient was 9% for UM and increased to 16% for PESG and 19% for PESP membranes. Similar improvement was observed for dextrans. For example, the R value for 110 kDa dextran increased from 50% for UM, to 66% for PESG and 73% for PESP. The differences in the separation efficiency of the 500 kDa dextran through the various membranes were smaller. The rejection coefficient amounted to 85%, 93%, and 95%, respectively. The better separation properties of the PESP membrane compared to the PESG one corresponds well with the permeance (Fig. 8).

Regardless of the casting dope composition, the incorporation of the Ag-TNTs improved the separation properties of the membranes. However, the improvement was more pronounced in the case of UM and PES/Ag-TNT than in the case of the membranes containing the pore-forming agents. The rejection coefficient increased from 9 to 20% for 20 kDa PEG, from 50 to 66% for 110 kDa dextran and from 85 to 94% for 500 kDa dextran. The increase in R value in the case of the PESP/Ag-TNT and PESG/Ag-TNT compared to PESP and PESG did not exceed 9 p.p. for 20 kDa PEG, and 6 p.p for 110 kDa and 500 kDa dextrans.

### Antifouling properties

**Fig. 9:**
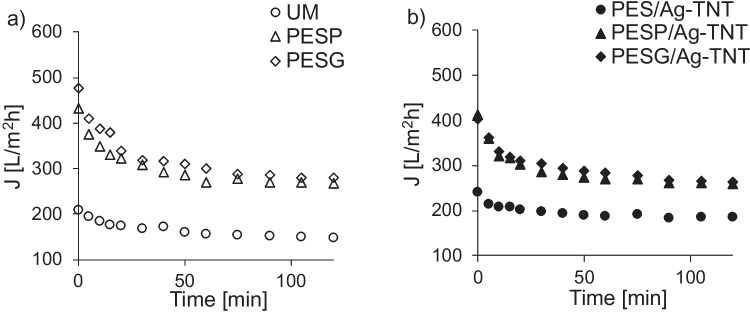
Effect of Ag-TNTs and pore-forming agents on BSA fouling of the PES membranes: **a** neat membranes, **b** membranes with Ag-TNTs. Initial BSA concentration: 1 g/L; TMP = 2 bar

The membrane fouling was investigated using BSA as a model substance. The results are presented in Fig. 9.

It can be observed that the membranes modified with porogens were characterized by higher permeate fluxes compared to the neat UM. That reflects the higher water permeability of these membranes (Fig. 9). Nonetheless, regardless of the membrane type, a deterioration of the flux in time was observed. The most pronounced decrease took place in the initial 30 min of filtration, after that the flux got stabilized, and ca. 35% of permeate flux decline was noted in case of both modified membranes (PESP/Ag-TNT and PESG/Ag-TNT) after 2 h of ultrafiltration of BSA (Fig. 9b). Such a phenomenon is related to the mechanism of membrane fouling by BSA. The foulant molecules are easily attracted by the negatively charged membrane surface, and the interactions are the strongest at the beginning of filtration, which corresponds to the highest flux decrease (Zhang et al. 2020). At this stage of the process the membrane surface is covered with a thin uniform layer of the rejected substance. As a result the membrane changes its charge into more positive, which reduces the interactions between the membrane surface (already fouled) and BSA molecules remaining in feed. This eventually leads to the stabilization of permeate flux (Li et al. 2016). The gel layer formed onto membrane surface was mainly responsible for noticed flux during ultrafiltration of BSA (Mokarizadeh and Raisi 2021). The same authors observed a gradual decrease of permeate flux with increasing of concentration of PEG or PVP from 5 to 15% as a result of more dense structure of prepared membranes (Mokarizadeh and Raisi 2021).

The data presented in Fig. 9 revealed that application of PES and PEG resulted in a deterioration of the fouling resistance of the membranes. At the end of filtration the permeate flux was lower compared to pure water flux (PWF) by 57% in the case of UM, by 62% in the case of PESP and by 64% in the case of PESG. The incorporation of Ag-TNTs to the membranes modified with porogens resulted in a further aggravation of the fouling resistance. After 120 min of filtration the flux was lower by 71–72% compared to PWF. On the opposite, the addition of the NPs to the neat PES membrane led to fouling mitigation, as the decrease in the flux was less severe (49%) compared to the neat UM.

**Fig. 10:**
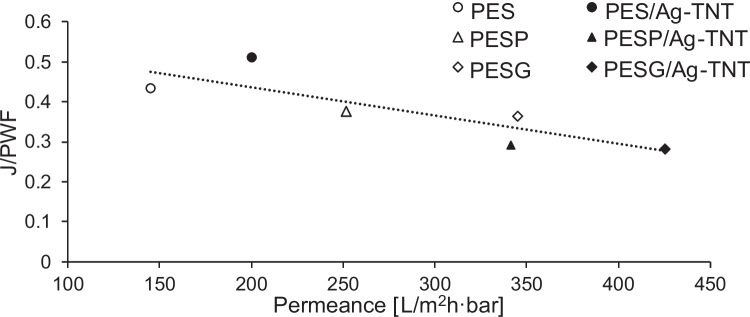
Influence of water permeance on the permeate flux decline (J/PWF) during filtration of BSA solution (1 g/L). The dashed line is a guide to the eye

The lower fouling resistance of the membranes containing pore formers can be attributed to their higher water permeability. Figure 10 shows the effect of water permeance on the fouling resistance of the membranes.

It can be observed that the membranes characterized by a higher PWF (PESP, PESG, PESP/Ag-TNT, PESG/Ag-TNT) were more prone to fouling compared to the membranes with lower permeability (UM, PES/Ag-TNT). The higher permeability results in an intensified mass transport towards the membrane surface which causes deposition of more albumin molecules (Gebru and Das 2017). As a result a thicker and denser BSA layer is formed, leading to a more severe permeate flux decline. The higher proneness of the membranes containing porogens to BSA fouling can be also attributed to their higher roughness compared to the UM and PES/Ag-TNT membranes (Figs. 3 and 4). The presence of deep valleys on the PVP and PEG-based membranes surface created the advantageous conditions for deposition of BSA molecules, which resulted in fouling enhancement. Thus, the observed results could be caused by the electrostatic interactions of BSA and membrane surface with nanoadditives, as well as hydrophilicity and surface structure (Jaber et al. 2024).

## Antibacterial properties

**Fig. 11:**
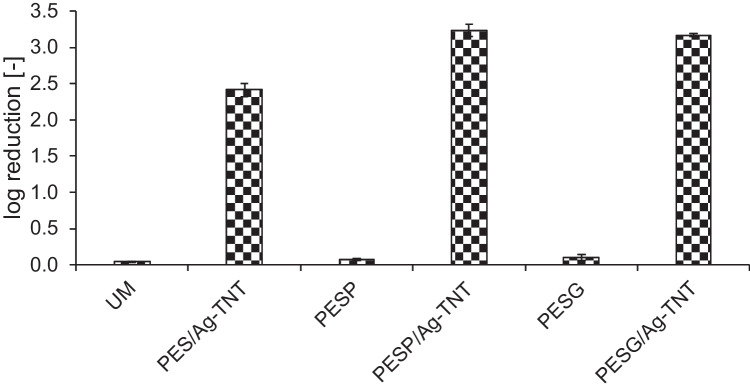
Influence of the pore-forming agents and Ag-TNTs on antibacterial properties of PES membranes against *E. coli*. Experimental conditions: stirred system, mixing at 200 rpm for 24 h, temperature: 37 °C

Antibacterial properties of the membranes were investigated in two systems. In the first one a piece of a membrane was incubated in the *E. coli* suspension under stirring conditions, while in the second one a cross flow filtration of the bacterial suspension was conducted. Figure 11 shows the results collected in the stirred system.

Similarly as the UM membrane, also the PESP and PESG membranes did not exhibit any significant antibacterial activity. The log reduction value did not exceed 0.1, which corresponds to about 20% inhibition of bacterial growth. The observed results can be attributed to the cell damage due to the stirring of the *E. coli* suspension during the experiment. After the introduction of Ag-TNTs into the membranes a noticeable increase in antibacterial properties was observed. The log reduction for the PES/Ag-TNT membrane was 2.4, and was lower compared to the PESP/Ag-TNT (3.2) and PESG/Ag-TNT (3.2). Nonetheless, in the case of all NPs-modified membranes the inhibition of bacterial growth exceeded 99.6%. The better antibacterial properties of the membranes modified with Ag-TNTs and PEG/PVP compared to the membrane containing NPs without the addition of the porogens can be attributed to the hydrophilic properties of the modifying agents. During the phase inversion, the presence of the hydrophilic additives favors the transport of NPs towards the skin layer of the membrane, and therefore, the Ag-TNTs can more efficiently interact with microorganisms (Andrade et al. 2015). In the case of the hybrid NPs applied in the present study the antibacterial mechanism is complex and arises from the mechanical damage of the cells by TNTs protruding from the membrane (Mozia et al. 2021) as well as from the antimicrobial properties of silver. The antibacterial action of Ag is commonly explained by three main mechanisms: (i) Due to the high affinity of silver to sulfur, silver nanoparticles have the ability to attach to the cell membrane causing structural and functional changes in it (Zhang and Chen 2009). (ii) Silver ions released from the NPs due to the oxidation process can combine with nucleic acids, leading to DNA condensation and loss of replication ability. In addition, silver ions, due to their high affinity to the thiol group, disrupt the respiratory chain, which ultimately leads to cell damage (Zhang and Chen 2009). (iii) In the presence of Ag NPs various reactive oxygen species (ROS) can be formed, including H_2_O_2_, ^•^OH or O_2_^–•^. These ROS also contribute to the antibacterial action of the NPs (Ouay and Stallecci 2015). Any changes in cell structures can lead to disturbances in the functioning of bacteria, leading to their death.

**Fig. 12:**
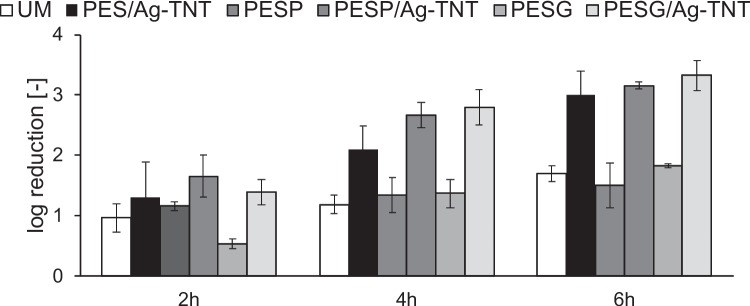
Inhibition of *E. coli* growth on feed side during ultrafiltration of the bacterial suspension. Process conditions: filtration system, transmembrane pressure: 1 bar, feed cross flow velocity: 0.25 m/s, temperature: 37 °C

In the second part of the study on the antimicrobial properties of the membranes the ultrafiltration of *E. coli* suspension was conducted for 6 h. Figure 12 shows the efficiency of the inhibition of bacterial growth determined on a basis of feed and retentate composition (i.e., retention by the membrane was not considered in this calculation).

It can be observed that the antibacterial activity of the Ag-TNTs-modified membranes was visibly higher compared to the membranes without NPs addition. That confirms the results obtained in the first stage of the study. However, in contrast to the results shown in Fig. 11, the differences between the inhibition of bacterial growth in the presence and absence of Ag-TNTs were lower (Fig. 12). It was also found that the value of log reduction increased in time, regardless of the membrane. Eventually, after 6 h of filtration the log reduction in the case of the membranes without NPs ranged from 1.5 to 1.8, while in the presence of Ag-TNTs it amounted to 3.0–3.3. The observed higher inhibition of bacterial growth in the filtration system compared to the stirred one, especially for the membranes not containing NPs, can result from more aggressive conditions in the UF installation. The applied transmembrane pressure (1 bar), as well as feed cross flow velocity (0.25 m/s) affected the number of bacteria cells in the system. Nonetheless, the data shown in Fig. 12 prove the positive influence of Ag-TNTs on the antibacterial activity of the PES membranes. The decrease in the cell viability and death of the bacteria due to the presence of nanoadditives on the membrane surface indicates the formation of non-tolerant surface for bacteria survival (Salim et al. 2022). The antibacterial properties of the composite membranes could be associated with the hydrophilic nature of the nanoadditives into membrane matrix. The presence of hydrophilic groups in the polymeric matrix and the hydrophilic nature of the Ag-TNTs at the surface of membrane result in bacteria death, owing to the progress of cell lysis (Salim et al. 2022).

During the UF process, the retention of *E. coli* by the membranes was also monitored. The retention coefficient for all the membranes exceeded 97%. In the case of the unmodified membrane, the retention after 6 h was 99.64%, and after the introduction of Ag-TNTs, it increased to 99.88%. The R value for the PESP and PES/Ag-TNT membranes reached 99.44 and 99.53%, respectively, and for the PESG and PESG/Ag-TNT membranes, 99.93 and 98.90%, respectively. The obtained results show that even incorporation of the biocidal NPs in the membrane matrix did not allow for the production of microbiologically safe permeate, since some single bacterial cells were still detected in the product. There may be various reasons for this phenomenon (Lebleu et al. 2009). It was proved that the size and shape of bacterial cells are not the key parameters deciding about their transport or rejection by MF membranes. For example, *Corynebacterium xerosis* having similar size to *E. coli*, were efficiently rejected by a membrane, while the latter bacteria were transported through the pores with a diameters lower than the bacteria dimensions. Furthermore, *Staphylococcus aureus*, despite smaller dimensions compared to *E. coli*, was rejected with a higher efficiency. As a result, it was concluded that the sieve effect is not the only mechanism of separation in this case, and there are some other factors, e.g., surface charge or hydrophobicity, which affect separation of microorganisms. The possibility of the presence of defects in the structure of the surface layer of the membranes, e.g., due to the formation of interconnected larger pores during phase inversion, was also proposed as a reason for the passage of bacteria thorough the membranes. Nonetheless, in the filtration system applied in the current study, the presence of bacterial cells in the permeate may be mainly related to the process conditions. The stress created during the UF process can lead to a reduction in volume and deformation of the surface of the bacteria which could allow the cell to penetrate into the pores of the membrane. Under the action of shear and drag forces due to the feed cross flow and transmembrane pressure applied, the cells can reduce their volume and change shape, which allows their passage through a membrane. Such a mechanism is especially related to the Gram-negative bacteria which are characterized by a thin peptidoglycan layer, and thus have a tendency to deform and penetrate pores smaller than their original size (Lebleu et al. 2009). Some authors emphasized the role of concentration of Ag in the membrane matrix (Ihsanullah et al. 2015). According researchers, the amount of silver increasing from 10 to 20% significantly slowed down the killing effect as a result of Ag aggregates and thus limiting the contact of silver nanoparticles with cells of *E.coli* (Ihsanullah et al. 2015).

**Fig. 13:**
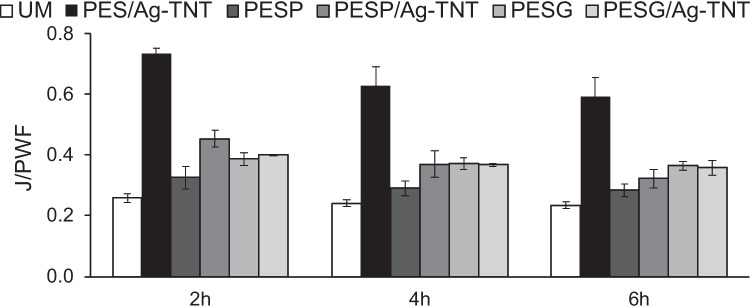
Influence of pore-forming agents and Ag-TNTs on permeate flux during ultrafiltration of *E. coli* suspension. Process conditions: transmembrane pressure: 1 bar, feed cross flow velocity: 0.25 m/s, temperature: 37 °C

Figure 13 shows changes in permeate flux during ultrafiltration of *E. coli* suspension.

**Fig. 14:**
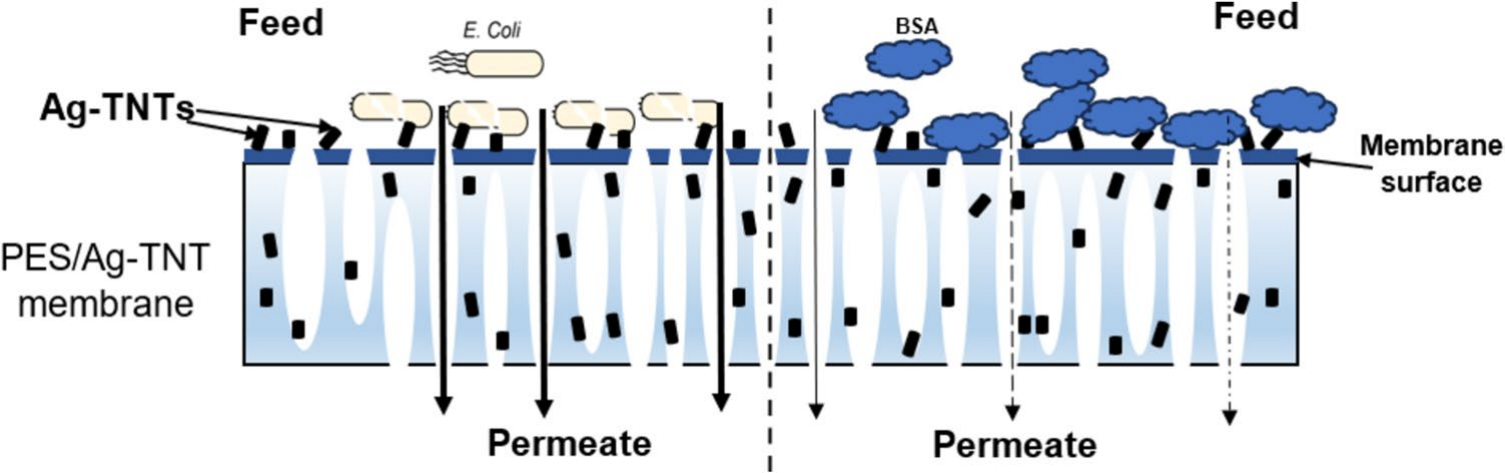
Mechanism of flow through the membrane and the anti(bio)fouling of membranes

It was found that the most significant deterioration of the flux took place in the initial 2 h of filtration. Compared to PWF, the permeate flux decreased by 74%, 67%, and 61% for UM, PESP and PESG, respectively, while after 6 h the flux was lower by 77%, 72%, and 63%. In the case of the PES/Ag-TNT, PESP/Ag-TNT, and PESG/Ag-TNT membranes the flux decreased after 2 h of filtration by 27%, 55%, and 60%, respectively, and at the end of the experiment the decline by 41%, 68%, and 64% was observed. It was found that the application of the pore-forming agents in the absence of the NPs improved the membrane resistance to fouling by bacterial cells compared to UM. After 6 h of filtration the decrease in permeate flux for PESP and PESG membranes was less severe than for UM membrane. The incorporation of Ag-TNTs improved the antifouling properties especially in the case of PES/Ag-TNT (Fig. 13). The permeate fluxes measured for the PESP/Ag-TNT and PESG/Ag-TNT membranes were similar to the corresponding neat PVP and PEG-containing membranes. In the Fig. 14, a scheme of flow through the membrane and anti(bio)fouling mechanism was presented.

**Fig. 15:**
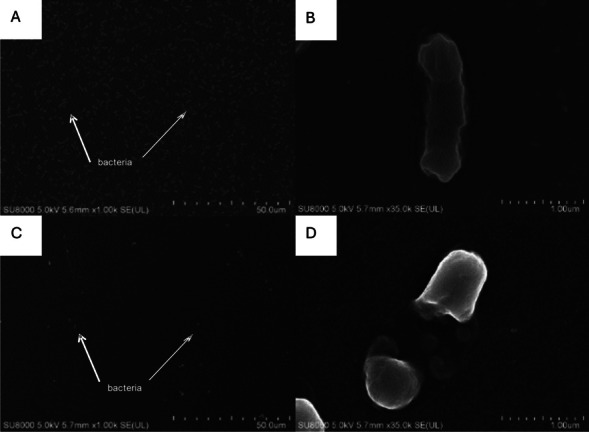
Membrane surface covered by *E. coli* at the beginning of the process and after the experiment (**A**, **C**). *E. coli* cell before and after contact with Ag-TNTs presence onto membrane surface (**B**, **D**)

The visibly higher antibiofouling performance of the PES/Ag-TNT can be attributed to its lower roughness compared to the membranes containing PVP and PEG. Nonetheless, the presence of pore formers improves membrane hydrophilicity and thus can reduce the deposition of the bacterial cells on the membrane surface. However, although microbial cells play an important role in biofouling, the decrease in flux is also influenced by the accumulation of extracellular polymeric substances (EPS). They facilitate the deposition of bacterial cells on a membrane surface and stimulate the growth of bacteria, which results in biofilm development (Fonseca et al. 2007). The rough surface of the PVP and PEG-modified membranes can enhance the accumulation of EPS, and the deposited material can form a gel layer on the membrane surface. In the presence of Ag-TNTs the damage of the bacterial cells due to the action of silver and nanotubes occurs, what results not only in a limitation of *E. coli* growth but also in a release of the cell content. In the Fig. 15, there is the surface of membrane covered by *E. coli* at the beginning of the process and after the experiment. Significant reduction of bacteria cells is visible (Fig. 15A and C). In the Fig. 15B and D, the *E. coli* cell before and after contact with Ag-TNTs presence onto membrane surface, are presented. It can be seen that the bacteria is damaged after acting of nanoadditive (Fig. 15 D).

These intracellular substances can additionally contribute to the decline of the permeate flux. Therefore, the combined influence of a high roughness, the presence of EPS and the release of intracellular substances leads to a lower antibiofouling performance of the PESP/Ag-TNT and PESG/Ag-TNT membranes compared to the PES/Ag-TNT. In the MBR system equipped with PES/PEG or PES/PVP membrane, a higher initial permeate flux was noted, after that, gradual decrease was observed by Nur-E Alam et al. (Nur-E Alam et al. 2024), what was related with a structure of prepared membranes.

## Conclusions

The application of PVP and PEG as porogens together with Ag-TNTs as antibacterial NPs influenced the physicochemical properties, morphology, permeability, as well as anti-(bio)fouling performance and antibacterial activity of PES membranes. The membranes modified with PVP or PEG revealed improved hydrophilicity and water permeability, however, the increased surface roughness was also observed. The introduction of Ag-TNTs further enhanced permeability, regardless of the type of the pore former, which was attributed to the tubular structure of the nanomaterial and its hydrophilic nature. A positive influence of the NPs on the improvement in separation properties of the membranes was also observed. The rejection of model organic molecules increased upon addition of Ag-TNTs, regardless of the porogen used. A negative influence of PEG or PVP application on the resistance of the PES membranes to BSA fouling was observed. That was attributed to a high permeability which enhanced the transport of the foulant to the membrane surface and its deposition on it. The incorporation of Ag-TNTs into the PESP and PESG membranes resulted in a further deterioration of the fouling resistance. That was explained by the increase in roughness which resulted in a reduction of the positive effect of the hydrophilic properties of the applied additives. Nonetheless, despite the reduced fouling resistance, the PVP or PEG-modified membranes still exhibited higher permeate fluxes during BSA filtration than neat UM before the modifications.

No significant influence of the type of porogen on the antibacterial properties of the neat PES membranes was observed. However, introduction of Ag-TNTs resulted in an enhancement of antimicrobial action of PESP/Ag-TNT and PESG/Ag-TNT membranes compared to the PES/Ag-TNT. A higher efficiency of the inhibition of *E. coli* growth was observed for the filtration system compared to the stirred one. That was attributed to a more aggressive conditions in the case of the process conducted in the UF system. It was found that even the application of the membranes containing antibacterial Ag-TNTs nanomaterial did not allow to obtain a product (permeate) microbiologically safe. Although the *E. coli* retention coefficient exceeded 97% for all the membranes, some single cells were observed in permeate. A positive influence of the pore formers on biofouling mitigation was found. However, in the case of mixed matrix membranes, a visibly better antibiofouling performance was observed for PES/Ag-TNT compared to PESP/Ag-TNT and PESG/Ag-TNT membranes. That was explained by a higher roughness of the latter membranes, which in the presence of extracellular and intracellular substances limited the positive effect of the applied modification. Nonetheless, the application of either single PVP or PEG porogen, single Ag-TNTs or both a porogen and Ag-TNTs resulted in an improvement in antibiofouling performance of the modified membranes compared to the neat PES membrane (UM).

## Data Availability

The datasets used and/or analysed during the current study are available from the corresponding author on reasonable request.

## Abbreviations

AFM: Atomic force microscope
Ag-TNTs: Titanate nanotubes modified with silver
BSA: Bovine serum albumin
C_f_: Concentration of model compound in feed
CFU: Colony forming unit
CNTs: Carbon nanotubes
C_p_: Concentration of model compound in permeate
Cu-TNTs: Titanate nanotubes modified with copper
DMF: *N**,N** –* Dimethylformamide
EPS: Extracellular polymeric substances
HNTs: Halloysite nanotubes
HPLC: High performance liquid chromatograph
MMMs: Mixed matrix membranes
N: Number of bacteria colonies visible on agar plates
NIPS: Non-solvent induced phase separation
NPs: Nanoparticles
PCA: Plate count agar
PEG: Poly(ethylene glycol)
PEI: Polyetherimide
PES: Polyethersulfone
PESG: Membrane with poly(ethylene glycol)
PESP: Membrane with polyvinylpyrrolidone
PSU: Polysulfone
PVDF: Polyvinylidene fluoride
PVP: Polyvinylpyrrolidone
PWF: Pure water flux
R: Rejection coefficient
R_a_: Mean surface roughness
RI: Refractive index
ROS: Reactive oxygen species
SCA: Static contact angle
SE: Secondary electrons
TMP: Transmembrane pressure
TNTs: Titanate nanotubes
UF: Ultrafiltration
UHR FE – SEM: Ultra-high-resolution field-emission scanning electron microscope
UM: Unmodified membrane
UV: Ultraviolet
Y: Dilution factor
Z: Volume of bacteria suspension on Petri dish
